# Sir2 Acts through Hepatocyte Nuclear Factor 4 to maintain insulin Signaling and Metabolic Homeostasis in *Drosophila*

**DOI:** 10.1371/journal.pgen.1005978

**Published:** 2016-04-08

**Authors:** Rebecca A. S. Palu, Carl S. Thummel

**Affiliations:** Department of Human Genetics, University of Utah School of Medicine, Salt Lake City, Utah, United States of America; Stanford University School of Medicine, UNITED STATES

## Abstract

SIRT1 is a member of the sirtuin family of NAD^+^-dependent deacetylases, which couple cellular metabolism to systemic physiology. Although studies in mouse models have defined a central role for SIRT1 in maintaining metabolic health, the molecular mechanisms remain unclear. Here we show that loss of the *Drosophila* SIRT1 homolog *sir2* leads to the age-progressive onset of hyperglycemia, obesity, glucose intolerance, and insulin resistance. Tissue-specific functional studies show that Sir2 is both necessary and sufficient in the fat body (analogous to the mammalian liver) to maintain glucose homeostasis and peripheral insulin sensitivity. Transcriptional profiling of *sir2* mutants by RNA-seq revealed a major overlap with genes regulated by the nuclear receptor Hepatocyte Nuclear Factor 4 (HNF4). Consistent with this, *Drosophila* HNF4 mutants display diabetic phenotypes similar to those of *sir2* mutants, and protein levels for dHNF4 are reduced in *sir2* mutant animals. We show that Sir2 exerts these effects by deacetylating and stabilizing dHNF4 through protein interactions. Increasing *dHNF4* expression in *sir2* mutants is sufficient to rescue their insulin signaling defects, defining this nuclear receptor as an important downstream effector of Sir2 signaling. This study demonstrates that the key metabolic activities of SIRT1 have been conserved through evolution, provides a genetic model for functional studies of phenotypes related to type 2 diabetes, and establishes HNF4 as a critical downstream target by which Sir2 maintains metabolic health.

## Author Summary

The sirtuins are a highly conserved family of deacetylases with targets that range from DNA-associated histones to transcription factors. The activities of these enzymes are dependent upon the energetic state of the cell as they utilize the coenzyme NAD^+^, an important electron carrier in central metabolic pathways. We have found that loss of the *Drosophila* homolog of the founding member of the sirtuin family, *sir2*, leads to age-progressive metabolic disease with symptoms similar to those of type 2 diabetes. In addition, we show that the *Drosophila* HNF4 nuclear receptor is deacetylated and stabilized by Sir2, and that it accounts for a major part of the transcriptional program controlled by Sir2. This work provides a new genetic model of insulin resistance in *Drosophila* and establishes HNF4 as a critical downstream target in the Sir2 signaling pathway.

## Introduction

The incidence of complex metabolic disorders has been on the rise for the past three decades, comprising an epidemic of ever-increasing severity. Much of this can be attributed to an increase in the prevalence of type 2 diabetes accompanied by insulin resistance, the development of which is complex and poorly understood. These trends have prompted widespread changes in public policy and a shift in biomedical research toward improving our understanding of the genetic and environmental factors that contribute to insulin resistance and its progression to a more severe disease state.

One focus for these studies has been the sirtuin family of NAD^+^-dependent deacetylases, which play a central role in coupling metabolic state to systemic physiology. Sirtuin activity is dependent upon the availability of NAD^+^, an important electron carrier that contributes to cellular redox balance, drives mitochondrial oxidative phosphorylation, and acts as an important enzymatic cofactor [1–4]. The founding member of the sirtuin family, yeast Sir2, was discovered based on its role in heterochromatin formation [5–7]. Subsequent studies of the mammalian Sir2 homolog, SIRT1, have defined it as a critical regulator of metabolic homeostasis, acting through multiple protein targets [1–4]. These include Foxo, the nuclear receptors PPARα and LXR, and co-activators such as PGC-1α [8–11]. The multiple downstream targets of SIRT1, combined with its dependence on NAD^+^, establish it as a pivotal energy sensor that couples cellular redox state to metabolic control.

Given the complexity of SIRT1 regulation and function, it is not surprising that genetic studies of SIRT1 in mice have been complicated by environmental factors and genetic background effects, occasionally leading to contradictory results [1, 3]. This has been most evident in the context of aging, where the beneficial effects of SIRT1 remain controversial. In contrast, tissue-specific functional studies of SIRT1, combined with overexpression experiments and pharmacological activation, have established important roles for this factor in maintaining metabolic homeostasis [1, 3, 4]. These include a role in pancreatic beta-cells to promote glucose-stimulated insulin secretion (GSIS) and improve glucose tolerance, as well as activities in peripheral tissues that promote insulin sensitivity [12, 13]. SIRT1 also supports fatty acid oxidation and oxidative phosphorylation in the liver, suppresses hepatic steatosis, and acts in white adipose to suppress lipid accumulation [9]. Conversely, low-level SIRT1 overexpression promotes glucose tolerance, insulin sensitivity, and prevents fatty liver disease, highlighting the beneficial effects of SIRT1 action and supporting the proposal that SIRT1 activation could be of therapeutic value [14–16]. In spite of these advances, however, the molecular mechanisms by which SIRT1 maintains metabolic homeostasis remain unclear [2].

Studies of the *Drosophila* SIRT1 homolog, Sir2, have recently begun to provide a better understanding of its roles in systemic physiology. Null mutants for *sir2* display increased levels of stored lipid, analogous to the role of SIRT1 in suppressing obesity [17, 18]. Elevated free glucose levels were also observed in mutant adults, accompanied by starvation sensitivity [18]. Other metabolic functions for *sir2*, however, have been based on overexpression experiments and RNAi [18, 19]. One of these studies reported that glucose levels are reduced in animals with ubiquitous RNAi against *sir2*, contradicting their data from mutants [18]. The RNAi studies also resulted in only a two-fold reduction in *sir2* expression, leaving it unclear how these results relate to gene function [18, 19]. These concerns, combined with the importance of genetic background on SIRT1 activities, led us to undertake a detailed metabolic analysis of a transheterozygous combination of *sir2* null alleles compared to genetically-matched controls. We show here that loss of *sir2* leads to the age-progressive development of obesity, hyperglycemia, glucose intolerance, and insulin resistance. Tissue-specific RNAi and genetic rescue experiments show that Sir2 function is both necessary and sufficient in the fat body to maintain insulin sensitivity. In addition, our studies show that Sir2 maintains insulin signaling through deacetylation and stabilization of the *Drosophila* ortholog of HNF4A, dHNF4. Sir2 interacts with dHNF4, dHNF4 levels are reduced in *sir2* mutants, and expressing wild-type dHNF4 restores insulin signaling in a *sir2* mutant background. Taken together, our results define dHNF4 as a key downstream target of Sir2 and provide insights into the molecular mechanisms by which Sir2 promotes insulin sensitivity and metabolic health.

## Results

### *sir2* mutants develop age-progressive symptoms of diabetes

**Fig 1:**
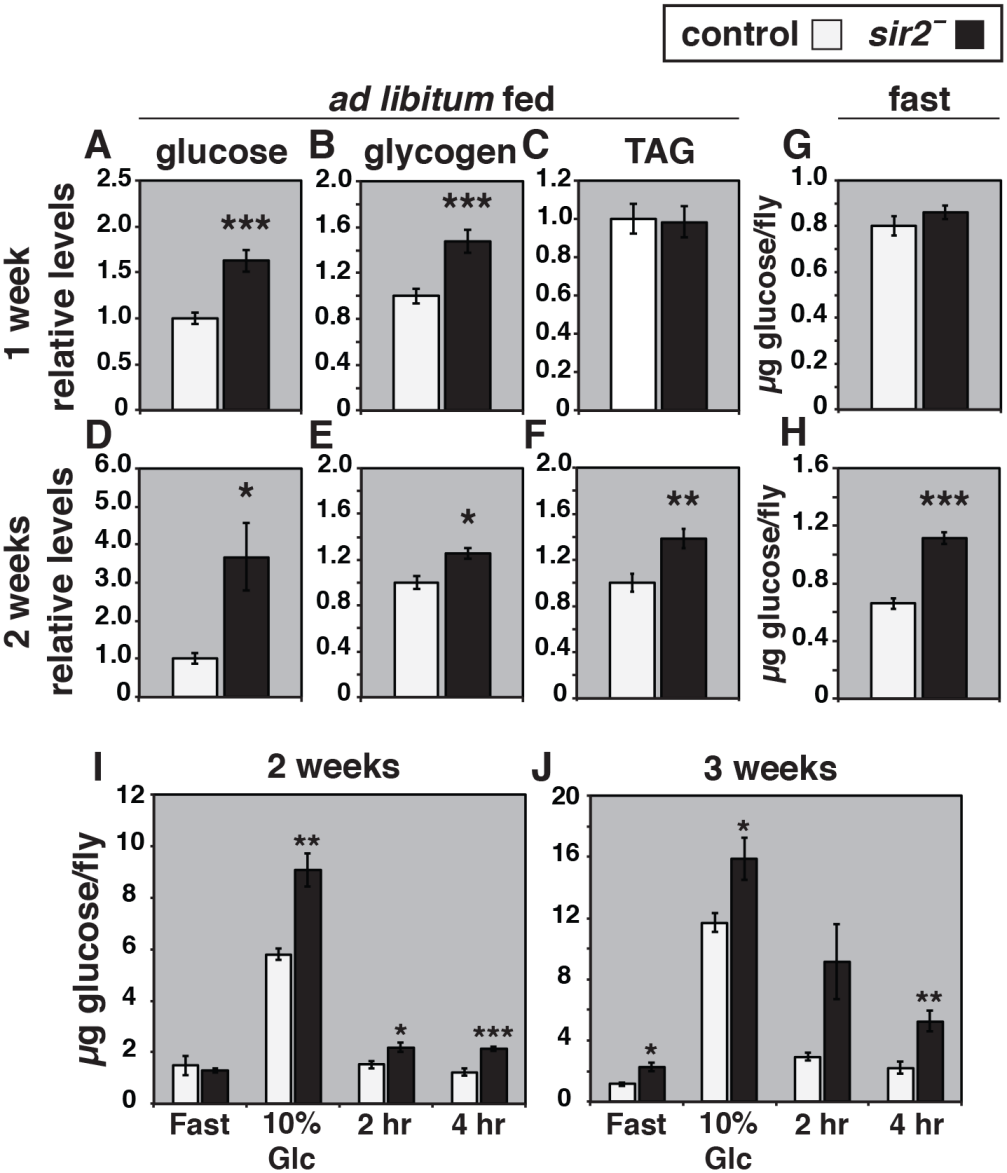
*sir2* mutants display age-dependent obesity and fasting hyperglycemia. Glucose (A,D), glycogen (B,E), and triglyceride (TAG) (C,F) levels were measured in control (white bars) and *sir2* mutants (black bars) at one (A-C) or two (D-F) weeks of age (n = 5–15 for each group). All values are normalized to soluble protein levels. (G-H) Glucose levels were measured in control (white bars) and *sir2* mutants (black bars) after an overnight fast, at one (G) or two weeks of age (H), and normalized to the number of animals (n = 37–43 for each group). An oral glucose tolerance test was performed on two (I) or three week (J) old controls (white bars) or *sir2* mutants (black bars). Animals were fasted overnight, fed on 10% glucose, re-fasted for either 2 or 4 hours, and free glucose levels were measured from whole animal homogenates (n = 5–10 for each group). Although *sir2* mutants do not display fasting hyperglycemia at two weeks of age in panel I, this is an exceptional result that was included because the overall profile of glucose clearance in this experiment best reflects our results from four independent replicates of this glucose tolerance test. The other three assays show fasting hyperglycemia, as depicted in panel H. Error bars are ± SEM. *p<0.05, **p<0.005, ***p<0.0005.

Two previously described deletion alleles of *sir2*, *sir2*^*2A-7-11*^ and *sir2*^*4*.*5*^, were used in transheteroallelic combination and compared to genetically-matched controls for all studies [20, 21]. As expected, *sir2* is not expressed in these mutants as assayed by RNA-seq or northern blot hybridization, consistent with their characterization as null alleles (S1A and S1B Fig). Unless otherwise indicated, adult male flies were used in all experiments, where the age indicated in the figures refers to the number of weeks after eclosion from the pupal case. The *sir2* mutants survive to adulthood and develop starvation sensitivity as previously reported (S1C and S1D Fig) [18, 21]. Basic metabolite measurements, however, reveal that they also display increasing metabolic dysfunction with age in the absence of significant effects on feeding rate (Figs 1 and S1E). At one week of age, *sir2* mutants have elevated levels of both free and circulating glucose as well as glycogen but no significant change in triglycerides (TAG) (Fig 1A–1C; S1F Fig). Elevated glucose and glycogen levels are still present at two weeks of age, but are also accompanied by elevated TAG, which is consistent with the increased lipid levels reported for *sir2* mutants (Fig 1D–1F) [17, 18]. In addition to this obesity, mutants at two weeks of age, but not one week, display fasting hyperglycemia, a hallmark of diabetes (Fig 1G and 1H). This is consistent with the results of metabolomic analysis of *sir2* mutants at two weeks of age, which revealed increased levels of glycolytic intermediates, including glucose-6-phosphate, dihydroxyacetone phosphate, and lactate (S2 Fig). Alternative glucose metabolites also increase significantly, such as the glucose alcohol sorbitol, which can accumulate to high levels in diabetics and may contribute to neuropathy and nephropathy [22].

An oral glucose tolerance test was used to determine if these age progressive defects in carbohydrate homeostasis can be accounted for by reduced peripheral glucose uptake. In this assay, male flies are fasted overnight and then allowed to consume 10% glucose for approximately one hour, after which they are transferred back to starvation media for either two or four hours. The kinetics with which they clear glucose from their systems is then monitored by performing glucose assays at each time point. The glucose levels in wild-type animals at both one and two weeks of age return to near fasting levels within two hours after glucose feeding (Fig 1I and 1J). Similarly, although *sir2* mutants at both two and three weeks of age are hyperglycemic after consuming glucose, they display relatively normal kinetics of subsequent glucose clearance at two weeks of age (Fig 1I). They are, however, clearly glucose intolerant by three weeks of age, as demonstrated by the continued high levels of glucose present after two hours of clearance on starvation media (Fig 1J). Taken together with our previous results, this indicates that *sir2* mutants display a progression of symptoms associated with a loss of glycemic control during early adulthood, from elevated levels of free glucose, to fasting hyperglycemia, to glucose intolerance.

### Insulin signaling defects arise in *sir2* mutants due to a loss of insulin sensitivity

**Fig 2:**
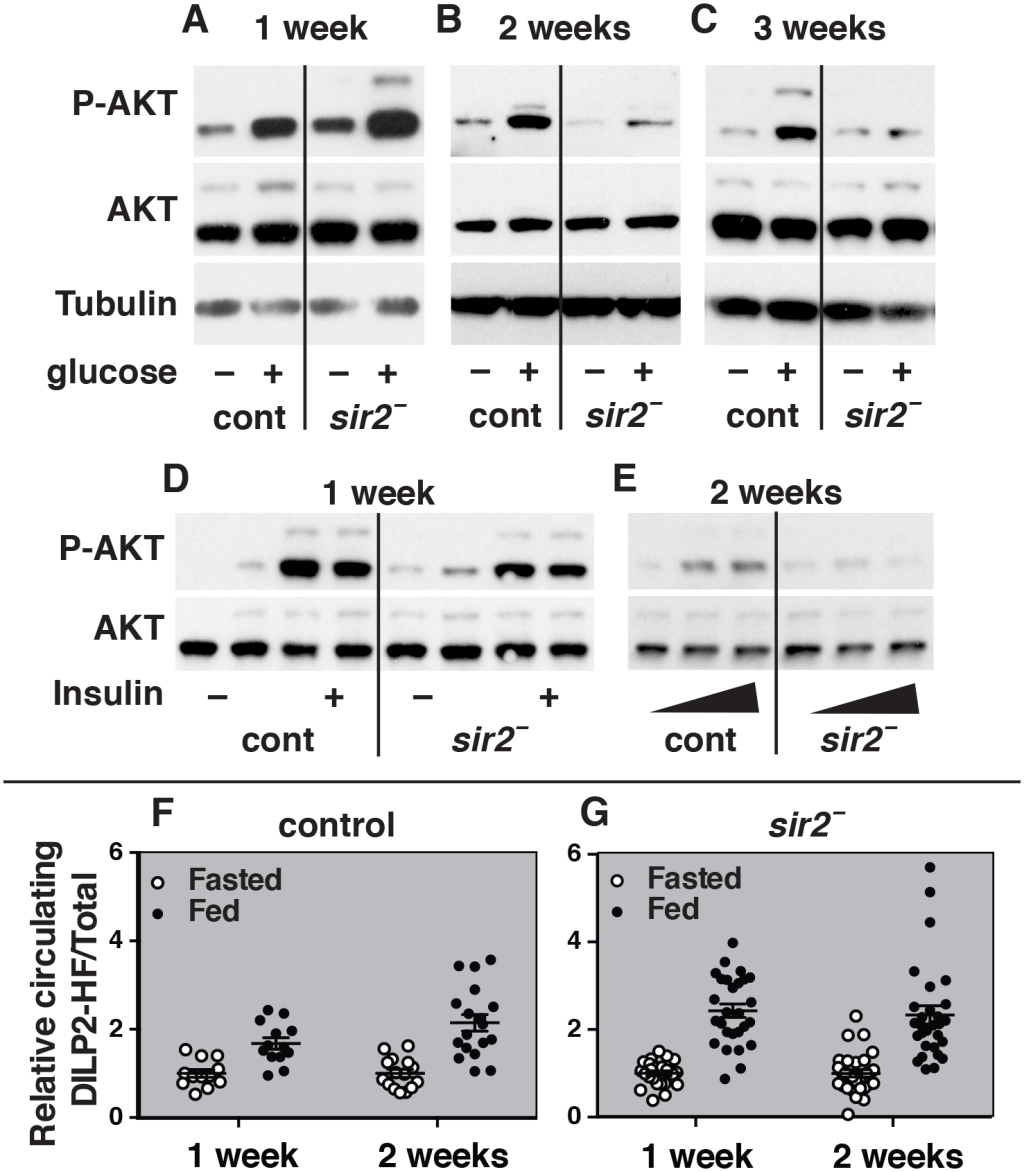
Loss of peripheral insulin signaling in *sir2* mutants is due to defects in insulin sensitivity. (A-C) Protein isolates from control (cont) and *sir2* mutant males (*sir2 –*) were analyzed on western blots using antibodies directed against phosphorylated AKT (P-AKT), total AKT, or Tubulin as a control. Animals were collected at one, two, or three weeks of age following a fasting-refeeding paradigm (–/+ glucose). The ratio of P-AKT levels to total AKT levels in refed controls and *sir2* mutants was quantified using data from three independent experimental replicates. The fold change between these ratios in mutants and controls is as follows, representing the mean ± SEM: (A) one week of age 0.8±0.3 (NS), (B) two weeks of age 0.2±0.06 (p = 0.005), (C) three weeks of age 0.1±0.08 (p = 0.008). (D,E) Insulin tolerance tests were performed at one (D) or two weeks (E) of age by comparing P-AKT levels after injection of bovine insulin (–/+ insulin, D; increasing insulin, E) following a fast. The ratio of P-AKT levels to total AKT levels in injected controls and *sir2* mutants was quantified using data from two biological replicates for each insulin concentration. The fold change between these ratios in mutants and controls is as follows, representing the mean ± SEM. (D) One week of age (- insulin) 3.3±1.5, (+ insulin) 0.7±0.01, two-way ANOVA between genotypes across insulin concentrations (NS). (E) Two weeks of age with increasing insulin concentrations, 1.0±0.6, 0.2±0.1, 0.3±0.2, two-way ANOVA between genotypes across insulin concentrations (p<0.05). (F,G) The amount of circulating DILP2 in controls (open circles) or *sir2* mutants (black circles) was determined using an ELISA assay following a fasting-refeeding paradigm. Each data point represents a single biological replicate (n = 10 flies/sample, n = 12–30 samples/group). Data is normalized to the fasting state for each experiment and presented as a scatter plot with the mean ±SEM indicated. Quantification of the fold-change in secreted DILP2 levels from the fasted to fed state is: one week controls: 1.0±0.09 to 1.7±0.1, two week controls: 1.0±0.2 to 2.2±0.2, one week *sir2* mutants: 1.0±0.07 to 2.5±0.2, two week *sir2* mutants: 1.0±0.08 to 2.7±0.4. In both controls and *sir2* mutants, there is a significant increase in DILP2 secretion in fed versus fasted animals (p<0.0001), but not between one and two weeks of age, as determined by two-way ANOVA. *sir2* mutants at one week of age have a stronger induction of DILP2 secretion in response to feeding than do controls (p = 0.008), while at two weeks the difference is not significant as determined by two-way ANOVA.

The development of diabetic phenotypes in *sir2* mutants with age could arise from a defect in peripheral insulin signaling. To determine if this is the case, we measured the levels of phosphorylated AKT (P-AKT), a downstream target of the insulin receptor, by western blot analysis of extracts from control and *sir2* mutants using a fasting/refeeding paradigm. While *sir2* mutants at one week of age respond normally to feeding by increasing their P-AKT levels, this response is reduced by two weeks of age and almost completely absent by three weeks of age (Fig 2A–2C). Consistent with this result, the Foxo target *4EBP* is incompletely repressed upon feeding in *sir2* mutants as compared to controls at two weeks of age (S3A Fig).

A decrease in insulin signaling could be due to a defect in either insulin sensitivity or insulin secretion. As expected, both controls and *sir2* mutants at one week of age have increased P-AKT in response to injected insulin, consistent with the activation of insulin signaling in response to dietary glucose (Fig 2A and 2D). In contrast, while control flies at two weeks of age continue to show increasing levels of P-AKT with increasing concentrations of injected insulin, *sir2* mutants fail to respond (Fig 2E). This indicates that *sir2* mutants are insulin resistant by two weeks of age.

We also measured secreted levels of *Drosophila* insulin-like peptide 2 (DILP2) in control and *sir2* mutants to determine if reduced DILP2 secretion could contribute to their defects in insulin signaling [23]. This study revealed that circulating DILP2 increases with age in both fasting and fed controls, and increases approximately two-fold in response to feeding (Fig 2F and 2G; S3B and S3C Fig), consistent with published wild-type responses [23]. Similar responses were seen in *sir2* mutants under these conditions at both one and two weeks of age (Fig 2F and 2G; S3B and S3C Fig). Taken together, these results indicate that defects in peripheral insulin sensitivity, but not insulin secretion, can account for the reduced insulin signaling in *sir2* mutants.

### Sir2 acts in the fat body to maintain insulin sensitivity and metabolic homeostasis

**Fig 3:**
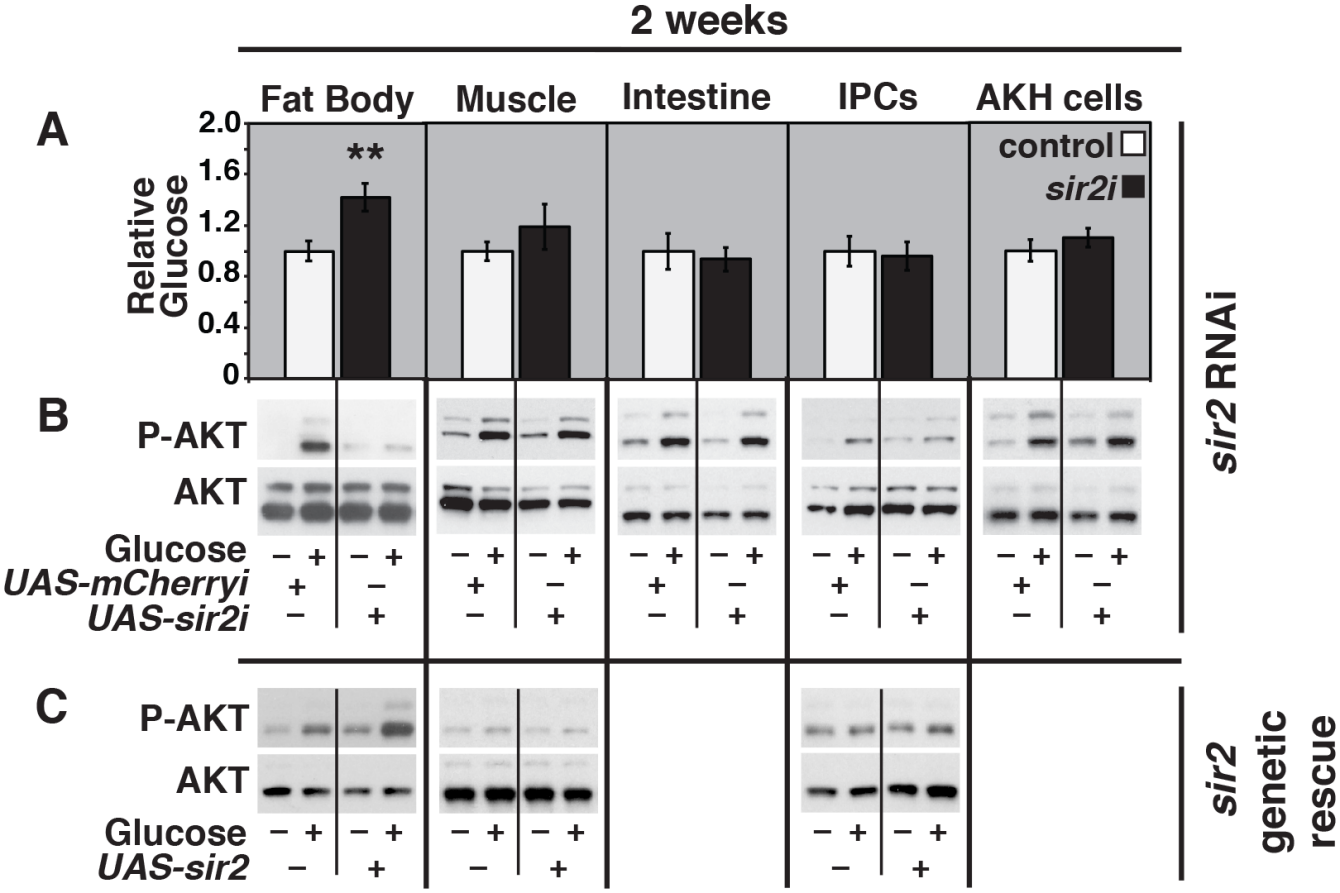
Sir2 acts in the fat body to regulate glucose homeostasis and insulin sensitivity. (A,B) Tissue-specific UAS-driven RNAi was directed against *mCherry* as a control (control, white bars) or *sir2* (*sir2i*, black bars) using the following GAL4 drivers: *r4-GAL4* (fat body), *mef2-GAL4* (muscle), *mex-GAL4* (intestine), *dilp2-GAL4* and *UAS-dcr2* (IPCs), or *AKH-GAL4* (AKH-producing cells). (A) Glucose levels were measured in two week old animals of each genotype and normalized to protein levels (n = 6–21 for each group, 5 males/sample). mean ± SEM is depicted. (B) Protein isolates from two week old animals of each genotype were analyzed on western blots using antibodies directed against phosphorylated AKT (P-AKT) or total AKT, following a fasting-refeeding paradigm (–/+ glucose). The ratio of P-AKT levels to total AKT levels in refed controls and *sir2* RNAi animals was quantified using data from three independent experimental replicates, except for *mef2-GAL4*, which had two replicates. The fold change between these ratios in *sir2* RNAi and control animals is as follows, representing the mean ± SEM: *r4-GAL4* 0.2±0.1 (p = 0.02), *mef2-GAL4* 1.7±0.5 (NS), *mex-GAL4* 0.9±0.2 (NS), *dilp2-GAL4>UAS-dcr2* 1.6±0.6 (NS), and *AKH-GAL4* 1.3±0.2 (NS). (C) Protein isolates were prepared from two week old *sir2* mutants carrying the following GAL4 drivers: *r4-GAL4* (fat body), *mef2-GAL4* (muscle), or *dilp2-GAL4* (IPCs), in either the absence or presence of a *UAS-sir2* rescue transgene (–/+ *UAS-sir2*). Equal amounts of protein were analyzed on western blots using antibodies directed against phosphorylated AKT (P-AKT) or total AKT, following a fasting-refeeding paradigm (–/+ glucose). The ratio of P-AKT levels to total AKT levels in refed controls (GAL4 driver/+ in *sir2*^*2A-7-11/4*.*5*^ mutants) and rescued *sir2* mutants (GAL4 driver/+, *UAS-sir2/+*, *sir2*^*2A-7-11/4*.*5*^) was quantified using data from two independent experimental replicates. The fold change between these ratios in *sir2* rescue and controls is as follows, representing the mean ± SEM: *r4-GAL4* 2.1±0.06 (p = 0.04), *mef2-GAL4* 0.9±0.2 (NS), *dilp2-GAL4* 0.9±0.1 (NS). **p<0.005.

The GAL4/UAS system was used to determine where Sir2 is necessary and/or sufficient to regulate metabolic homeostasis using tissue-specific RNAi and rescue experiments. Ubiquitous expression of a *sir2* RNAi construct efficiently eliminates *sir2* mRNA as assayed by northern blot hybridization, indicating that this approach provides a strong loss of *sir2* function (S4A Fig). Driving the expression of this construct in the fat body, but not the muscles, intestine, insulin producing cells (IPCs), or AKH-producing cells, disrupts insulin signaling and leads to hyperglycemia (Fig 3A and 3B). Consistent with this, tissue-specific expression of a wild-type *UAS-sir2* construct in the fat body of *sir2* mutants is sufficient to restore insulin signaling in peripheral tissues, with no rescue seen upon expression of *sir2* in the muscles or IPCs (Fig 3C). In addition, expression of *sir2* in the fat body, but not the muscle or IPCs, is sufficient to rescue the obesity of mutant animals, as reported previously (S4B Fig) [18]. Moreover, GAL4-driven expression of *sir2* in the fat body of wild-type animals is sufficient to reduce TAG levels, consistent with previous reports of *SIRT1* overexpression in mice (S4C Fig) [24]. These results define a central role for Sir2 in the fat body to regulate insulin signaling and suppress obesity and hyperglycemia. Given that the fat body performs functions analogous to the mammalian liver and white adipose tissue, these results are consistent with *Sirt1* studies in mice and suggest the *Drosophila* provides a valuable model to determine the molecular mechanisms by which this sirtuin promotes a healthy metabolic state.

### Sir2 regulates metabolic gene expression

As a first step to define the mechanisms by which Sir2 maintains metabolic homeostasis, we conducted RNA-seq analysis using quadruplicate RNA samples from control and *sir2* mutants at two weeks of age. A total of 400 genes were identified as differentially expressed in *sir2* mutants (≥1.5-fold change, *p*-value <0.05), with 312 genes down-regulated and 88 genes up-regulated (S1 Table). Gene ontology analysis revealed that the down-regulated genes are enriched in pathways related to the metabolic defects in *sir2* mutants, including proteolysis, lipolysis, carbohydrate metabolism, and genes involved in redox homeostasis, while many up-regulated genes are involved in *Drosophila* defense responses (S5 Fig) [25]. This could be analogous to the known role for Sirt1 in suppressing adipocyte inflammation and could contribute to the fat body-specific functions for *sir2* [26]. In addition, genes that are expressed at high levels in the intestine are enriched in the Sir2 down-regulated gene set, suggesting that this factor plays an important role in this tissue [27].

**Fig 4:**
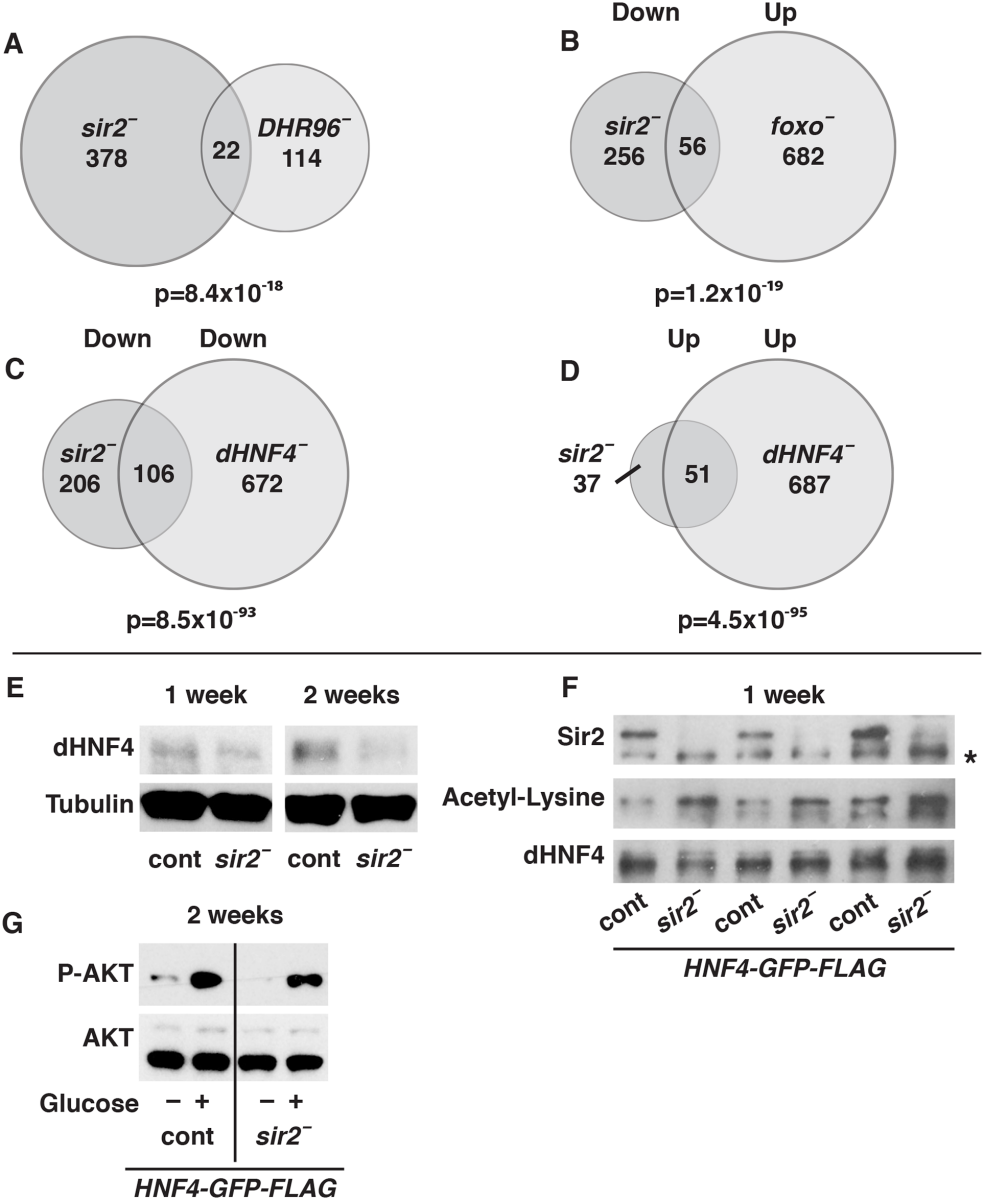
Sir2 acts through dHNF4 to maintain peripheral insulin signaling. Venn diagrams are shown depicting comparisons between (A) the genes regulated by *sir2* and *DHR96* mutants [28], (B) genes down-regulated in *sir2* mutants and up-regulated in *foxo* mutants [29], (C) genes down-regulated in both *sir2* and *dHNF4* mutants, and (D) genes up-regulated in both *sir2* and *dHNF4* mutants (W. Barry and C.S. Thummel, manuscript in revision). P-values were generated using a chi-square test, indicating the likelihood of the overlap by chance, given the number of genes in the *Drosophila* genome and the number of genes in each group. (E) Protein from control (cont) and *sir2* mutant (*sir2 –*) adult males were isolated at either one or two weeks of age and analyzed on western blots using antibodies against dHNF4 or tubulin as a loading control. The ratio of HNF4 protein levels to tubulin levels in each sample was quantified using data from three independent experimental replicates. The fold change between these ratios in *sir2* mutants and controls is as follows, representing the mean ± SEM: one week 0.7±0.01 (p = 0.0004), two weeks 0.3±0.03 (p = 0.002). (F) Protein extracts were prepared from controls (cont) or *sir2* mutants (*sir2*^*–*^) at one week of age carrying two copies of a genomic dHNF4 rescue construct tagged with GFP and FLAG (2X *HNF4-GFP-FLAG*). dHNF4 protein levels were normalized by loading a larger volume of *sir2* mutant lysates than controls after anti-FLAG immunoprecipitation. Extracts were analyzed on western blots using antibodies directed against Sir2, acetyl-lysine, or dHNF4. A background band detected by the Sir2 antibodies is marked (*). The ratio of acetylated HNF4 levels to total HNF4 levels in *sir2* mutants and controls was quantified using data from four independent experimental replicates. The fold change between these ratios in controls and mutants is 3.2±0.8 (p = 0.02), representing the mean ± SEM. (G) Protein isolates were prepared from controls (cont) and *sir2* mutants (*sir2*^*–*^) carrying two copies of the genomic dHNF4 rescue construct at two weeks of age, following a fasting-refeeding paradigm (–/+ glucose). These samples were analyzed on western blots using antibodies directed against phosphorylated AKT (P-AKT) or total AKT. The ratio of P-AKT levels to total AKT levels in refed controls and *sir2* mutants was quantified using data from four independent experimental replicates. The fold change between these ratios in controls and mutants is 2.1±1.1 (NS), representing the mean ± SEM.

Because transcription factors are prominent targets of Sirt1 regulation, we compared our RNA-seq dataset from *sir2* mutants with similar datasets for *Drosophila* transcription factors that control metabolism and insulin signaling. A small, but significant overlap is seen with genes regulated by the LXR homolog DHR96 in adults (15% of the 136 DHR96-regulated genes; Fig 4A), consistent with the known associations between Sirt1 and LXR [11, 28]. Similarly, we saw a significant overlap between genes that are expressed at reduced levels in *sir2* mutants and genes that increase their expression in *foxo* mutants (18% of the 312 *sir2*-down-regulated genes; Fig 4B) [29]. This is consistent with the decreased insulin signaling in *sir2* mutants as well as the known interactions between mammalian Sirt1 and Foxo [8]. Most remarkably, however, we saw a major overlap with genes regulated by the dHNF4 nuclear receptor, where more than 30% of the genes down-regulated in *sir2* mutants are also down-regulated in *dHNF4* mutants and nearly 60% of the genes up-regulated in *sir2* mutants are up-regulated in *dHNF4* mutants (Fig 4C and 4D) (W. Barry and C.S Thummel, manuscript in revision). This observation suggests that dHNF4 represents a major downstream target for Sir2 regulation in *Drosophila*.

### Sir2 interacts with dHNF4 and promotes its deacetylation

The simplest explanation for the large overlap between the genes regulated by *sir2* and *dHNF4* is that dHNF4 protein levels are reduced in *sir2* mutants. This is indeed the case as assayed by western blot, with an approximately 3-fold reduction in protein levels by two weeks of age, accompanied by a 1.7-fold reduction in *dHNF4* mRNA, with more mild effects in younger flies (Fig 4E; S6A Fig). Mammalian HNF4A can be regulated by acetylation, and lysines that are targets for this modification are conserved in *Drosophila* (S6B Fig) [30]. Consistent with this, when FLAG-tagged dHNF4 is immunoprecipitated and the levels of this protein are equalized between *sir2* mutants and controls, there is a 3-fold increase in the proportion of immunoprecipitated protein that is acetylated in *sir2* mutants (Fig 4F). Sir2 protein is also present in this immunoprecipitate, indicating that these factors interact physically (Fig 4F). Taken together, these results support the model that Sir2 interacts with dHNF4 to direct its deacetylation and maintain its stability.

### Expression of *dHNF4* in *sir2* mutants is sufficient to rescue insulin signaling

The reduced levels of dHNF4 protein in *sir2* mutants combined with the large overlap between the dHNF4 and Sir2-regulated gene sets suggests that Sir2 stabilizes and promotes the function of dHNF4. Consistent with this, ectopically increasing the levels of dHNF4 protein by crossing two copies of a genomic dHNF4-GFP-FLAG transgene into the *sir2* mutant background is sufficient to restore normal insulin signaling responses in these animals (Fig 4G). It is not sufficient, however, to rescue the hyperglycemia and elevated glycogen levels in *sir2* mutants (S6C and S6D Fig). We therefore conclude that some, but not all of the diabetic defects observed in *sir2* mutants are due to a reduction in dHNF4 levels.

## Discussion

Here we show that *sir2* mutants display a range of metabolic defects that parallel those seen in mouse *Sirt1* mutants, including hyperglycemia, lipid accumulation, insulin resistance, and glucose intolerance [1–3]. These results suggest that the fundamental metabolic functions of Sirt1 have been conserved through evolution and that further studies in *Drosophila* can be used to provide insights into its mammalian counterpart. An additional parallel with Sirt1 is seen in our tissue-specific studies, where we show that *sir2* function is necessary and sufficient in the fat body to maintain insulin signaling and suppress hyperglycemia and obesity, analogous to the role of Sirt1 in the liver and white adipose [9, 13, 24]. These results are also consistent with published studies of insulin sensitivity in *Drosophila*, which have shown that the fat body is the critical tissue that maintains glucose and lipid homeostasis through its ability to respond properly to insulin signaling [31, 32].

Our studies also define the dHNF4 nuclear receptor as a major target for Sir2 regulation. Consistent with this, *dHNF4* mutants display a range of phenotypes that resemble those of *sir2* mutants, including hyperglycemia, obesity, and glucose intolerance [33] (W. Barry and C.S. Thummel, manuscript in revision). As expected, these defects are more severe in *dHNF4* loss-of-function mutants, consistent with *sir2* mutants only resulting in a partial loss of dHNF4 protein. Sir2 interacts with dHNF4 and appears to stabilize this protein through deacetylation. This is an established mechanism for regulating protein stability, either through changes in target protein conformation that allow ubiquitin ligases to bind prior to proteasomal degradation, or through alternate pathways [34]. Further studies, however, are required to determine if this is a direct protein-protein interaction or part of a higher order complex.

Although two papers have shown that mammalian Sirt1 can control HNF4A transcriptional activity through a protein complex, only one gene was identified as a downstream target of this regulation, *PEPCK*, leaving it unclear if this activity is of functional significance [10, 30]. Our study suggests that this regulatory connection is far more extensive. The observation that one third of the genes down-regulated in *sir2* mutants are also down-regulated in *dHNF4* mutants (including *pepck*, S6A Fig), and most of the genes up-regulated in *sir2* mutants are up-regulated in *dHNF4* mutants, establishes this nuclear receptor as a major downstream target for Sir2 regulation. It will be interesting to determine if the extent of this regulatory connection has been conserved through evolution.

Despite this regulatory control, the over-expression of an HNF4 transgene was only able to partially restore the insulin signaling response and not the defects in carbohydrate homeostasis in *sir2* mutants. This lack of complete rescue is not surprising, given that the Sirt1 family targets a large number of transcription factors, histones, and enzymes, providing multiple additional pathways for metabolic regulation. Moreover, the activity or target recognition of dHNF4 may be altered when it is hyperacetylated, in which case merely over-expressing this factor would not fully restore normal function. Future studies can examine more direct targets, both previously characterized and uncharacterized, for their functions in suppressing diabetes downstream of Sir2-dependent regulation.

Finally, *sir2* mutants represent a new genetic model for studying the age-dependent onset of phenotypes related to type 2 diabetes. We show that newly-eclosed *sir2* mutant adults are relatively healthy, with elevated levels of free glucose and glycogen but otherwise normal metabolic functions. Their health, however, progressively worsens with age, with two-week-old *sir2* mutants displaying lipid accumulation, fasting hyperglycemia, and reduced insulin signaling accompanied by insulin resistance. This is followed by the onset of glucose intolerance by three weeks of age. Previous studies of type 2 diabetes in *Drosophila* have relied on dietary models using wild-type animals that are subjected to a high sugar diet [31, 32]. Although this is a valuable approach to better define the critical role of diet in diabetes onset, it is also clear that the likelihood of developing type 2 diabetes increases with age. The discovery that *sir2* mutants display this pathophysiology provides an opportunity to exploit the power of *Drosophila* genetics to better define the mechanisms that lead to the stepwise onset of metabolic dysfunction associated with diabetes.

## Materials and Methods

### Fly stocks and maintenance

Flies were raised at 25°C on media containing 8% yeast, 6% glucose, 3% sucrose, and 1% agar in 1XPBS for all studies, with flies maintained at 18°C for genetic rescue studies. Adult ages are indicated in the figures and text and refer to the time period after eclosion from the pupal case. Males under *ad libitum* feeding conditions were used for all experiments unless otherwise indicated. For most fasting-re-feeding paradigms, flies were transferred to 1% agar in 1XPBS for 14–18 hours and re-fed on 10% glucose, 1% agar in 1XPBS for two hours. A transheterozygous combination of the *sir2*^*2A-7-11*^ and *sir2*^*4*.*5*^ deletion alleles was used for all mutant studies [20, 21]. These alleles, all GAL4 lines (except for the *dilp2-gal4>UAS-dcr2* line), and the rescue construct, were outcrossed to a *w*^*1118*^ control strain, which was then used as a genetically-matched control for all experiments where indicated. The RNAi lines for *sir2* (#32481) and *mCherry* (#35787) were obtained from the Bloomington Stock Center. Immunoprecipitation experiments for dHNF4 were performed on lines containing a transgenic genomic construct with dHNF4 carrying GFP and FLAG tags, driven by the endogenous *dHNF4* promoter (Bloomington #38649). This transgene fully rescues *dHNF4* mutant defects and was maintained in homozygous *sir2*^*2A-7-11*^ or wild-type genetic backgrounds.

### Metabolite assays

Samples of five flies each were collected at one or two weeks of age and washed in 1XPBS. For triglycerides, glucose, glycogen, and protein, samples were homogenized in 120 μL of 1XPBS. For fasting glucose measurements, samples were homogenized in 100 μL trehalase buffer, and for ATP assays, samples were homogenized in 100 μL 6M guanidine HCl, 100mM Tris pH 7.8, 4mM EDTA). Assays were performed as described [35].

### Western blots

Samples of ten flies were collected under the indicated conditions at one, two, or three weeks of age, and homogenized in 100 μL of RIPA buffer containing 1X protease inhibitors (Roche cOmplete Mini EDTA-free protease inhibitor tablets). For P-AKT westerns, the buffer also contained Calyculin A and okadaic acid. Equivalent amounts of protein were resolved by SDS-PAGE (10% acrylamide), transferred to PVDF membrane overnight at 4°C, and blocked with 5% BSA prior to immunoblotting. Western blots were probed with antibodies for P-AKT (1:1000, Cell Signaling #4060), pan-AKT (1:1000, Cell Signaling #4691), Tubulin (1:5000, Abcam #ab184613), dHNF4 (1:1000–1:2000, generated by L. Palanker-Musselman), Sir2 (1:50, Developmental Studies Hybridoma Bank #p4A10), and pan-acetyl-lysine (1:1000, Cell Signaling #9441). The westerns shown in the figures are representative of at least three biological replicates. Quantification was performed by measuring protein levels using ImageJ software. The values reported represent the mutant or experimental condition normalized to the control, unless otherwise specified. For P-AKT quantification, the ratio of P-AKT levels to total AKT levels was determined in refed *sir2* mutants or the experimental condition and controls using ImageJ. The data from the fasted state was not quantified for these studies because the small changes in the basal levels of P-AKT under these conditions (ranging from undetectable to low levels) results in large statistical fluctuations that are not meaningful.

### RNA-seq

RNA was isolated from control and *sir2* mutants at two weeks of age using Trizol extraction (Thermo Fisher) and the Qiagen RNeasy Mini Kit. Library generation (Illumina TruSeq RNA Sample Preparation Kit v2 with oligo dT selection) and sequencing (HiSeq 50 Cycle Single Read Sequencing v3) were performed by the High-Throughput Genomics core facility at the University of Utah. The Bioinformatics Core Facility at the University of Utah aligned this dataset to the genome, utilizing the Genome Build DM3 from April 2006. Cut-offs for significance were Log2 ratio ± 0.585 and p-value <0.05 (<0.005 in all cases but two). RNA-seq data from this study can be accessed at NCBI GEO (accession number: GSE72947).

### Statistics

A standard two-tailed Student’s *t*-test was used to determine significance on basic metabolite measurements (Fig 1; S1F Fig). GraphPad PRISM 6 software was used to plot data and perform statistical analysis on all other measurements. Pairwise comparison p-values were calculated using a two-tailed Student’s *t*-test, and multiple comparison p-values were calculated using two-way ANOVA or Bonferroni correction. For metabolomics, p-values reflect a standard two-tailed unpaired t-test after a Welch’s correction for different variances. For the starvation sensitivity experiment, the p-values reflect results from both a Log-Rank Mantel-Cox test as well as a Gehan-Breslow-Wilcoxon test. For gene-regulatory overlaps, the p-values reflect results from chi-square tests.

### Cloning

The *UAS-sir2* rescue construct was generated by PCR amplification of the *sir2* coding region using primers designed to incorporate a KpnI restriction site in the forward primer (CGCG**GGTACC**CCAAATGGGTGCGAAGCTGACG) and an XbaI site in the reverse primer (CGCG**TCTAGA**GGCCCTCGGCTACGATTTCGCAG). The template for this reaction was cDNA generated from wild-type RNA using the ProtoScript M-MuLV *Taq* RT-PCR Kit (NEB). The gel-purified PCR product was digested with KpnI and XbaI and inserted into the multiple cloning site of pUAST-attB. This construct was integrated into each of three attP sites that are predicted to be silent (attP40, attP2, attP3) using standard methods (BestGene Inc.) [36]. We then combined this transgene with our *sir2*^*2A-7-11*^ allele in order to study its effect in a transheterozygous *sir2* mutant background. Only the rescue line inserted on the third chromosome at attP2, however, had sufficiently low background levels of *sir2* expression to allow us to see changes in triglyceride levels and insulin signaling using tissue-specific GAL4 drivers, as shown in Figs 3C and S4B. Background expression from the *UAS-sir2* transgene in all three lines is sufficient to rescue the hyperglycemia of *sir2* mutants, preventing us from examining the tissue-specific regulation of this response in Fig 3.

### Northern blots

RNA was isolated from samples containing 10–15 flies using Trizol (Thermo Fisher). Males were used for all studies except for the *Act>sir2-RNAi* experiment in S4A Fig, as only females were obtained from this cross. Northern blot transfers and hybridizations were performed as previously described [37].

### Feeding rate assays

Feeding rates were measured by using radioactive media containing ~5,000 cpm/μL α-^32^P-dCTP in 8% yeast, 6% glucose, and 3% sucrose in 1% agar. Male flies at one or two weeks of age were fasted overnight and then allowed to re-feed on the labeled media for two hours, after which they were transferred to unlabeled food for 45 minutes and sorted into samples of five flies on ice. A scintillation counter was used to measure the radioactivity in each sample, and this value was used to determine the relative volume of media consumed.

### Starvation sensitivity assays

Groups of 197–198 flies of each genotype, at two or three weeks of age, were transferred to fresh food for about 12–24 hours, and then transferred to starvation media. Lethality was monitored every four to eight hours, with surviving flies transferred to fresh starvation media at least once over the course of the experiment.

### Glucose tolerance test

Flies at two or three weeks of age were fasted overnight for 15–18 hours prior to re-feeding on 10% glucose. After one hour, flies were transferred back to starvation media for either two or four hours. Samples were collected at each time point for glucose assays, which were performed as described [35].

### Insulin tolerance test

Bovine insulin (Sigma) was dissolved in 1% acetic acid at 1 mg/mL before dilution to between 0.5 nM-100 nM in 1XPBS and 5% food dye. Flies were fasted overnight (one week old flies) or 4 hours (two week old flies) prior to insulin/dye injection in the thorax, until the dye was visible throughout the head and abdomen. Injections were performed for 10–15 minute intervals followed by an additional 30 minute rest period prior to collection of protein samples for western blot analysis. Concentrations at 1 week of age were 100 nM, and at 2 weeks of age were 0.5–1.0 nM [31, 38].

### Assays for circulating glucose

To extract hemolymph, 30 one week old flies were punctured in the thorax between the head and wing junction using a tungsten needle and centrifuged at 9,000xg for five minutes through a Zymo-Spin IIIC filter (Zymo Research). These samples were diluted 1:100 in Trehalose buffer and heat treated at 70°C for five minutes. Final dilutions of 1:200 and 1:400 were used for glucose assays.

### ELISA assay for circulating Dilp2

ELISA assays were performed as described on one and two week old flies [23]. Heterozygous control and homozygous *sir2*^*2A-7-11*^ mutant lines were established that contained two copies of the transgenic *dilp2* construct carrying HA and FLAG tags, driven by the genomic *dilp2* promoter in a *dilp2* mutant background (Dilp2-HF). Ten flies were collected per sample. Undiluted circulating Dilp2-HF was measured from hemolymph samples and total Dilp2-HF levels were measured at a 1:10 dilution.

### Metabolomics

Samples of fifteen adult males at two weeks of age were snap-frozen in liquid nitrogen and prepared for analysis by gas chromatography-mass spectrometry (GC/MS) as described [35]. Each experiment was performed on six independent samples, and each experiment was repeated three times. The data presented reflect the combined replicates from all three experiments, normalized within each experiment, for a total of 17–18 biological replicates per group. In one experimental replicate we failed to detect DHAP, for which there are only 12 biological replicates per group.

### Immunoprecipitation

Samples were collected from control and *sir2*^*2A-7-11*^ homozygous lines at one week of age containing two copies of the *dHNF4-GFP-FLAG* genomic transgene. Ten flies were homogenized in 100 μL homogenization solution consisting of RIPA buffer with protease inhibitors (Roche cOmplete Mini EDTA-free protease inhibitor tablets). Mouse anti-FLAG antibody (Sigma #F1804) was added to this homogenate at a 1:500 dilution and incubated for one hour, rotating at 4°C. A 1:1 mixture of Protein A/Protein G Dynabeads (Life Technologies) was washed with 1 mL RIPA three times before being resuspended in homogenization buffer. The equivalent of 10–20 μL of the original volume of washed beads was added to each homogenate and incubated for an additional two hours, rotating at 4°C. Immunoprecipitates were then eluted according to standard procedures in 1X sample buffer with protease inhibitors. 5–7.5 μL of the resulting elutes were loaded into a 10% SDS-PAGE gel Proteins were resolved by SDS-PAGE and analyzed on western blots as described above.

## Supporting Information

S1 Fig *sir2* null mutants are sensitive to starvation and hyperglycemic. (A) A gene model for *sir2* is shown, with the coding region in black and non-coding regions in gray. The regions deleted in the *sir2*^*2A-7-11*^ and *sir2*^*4*.*5*^ alleles are shown in green. Expression of *sir2* from controls (red) and *sir2* mutants (blue) was determined by RNA-seq analysis, with the reads assembled using an integrated genomics viewer (IGV). There is no measurable expression of the *sir2* coding region in transheterozygous mutants. (B) *sir2* transcripts are also not detectable in *sir2* mutants by northern blot hybridization, using *rp49* mRNA as a loading control. (C,D) The survival of *sir2* mutants (*sir2 –*) on starvation media is similar to that of controls (cont) at two weeks of age (C), but is significantly reduced at three weeks of age (D). (E) *sir2* mutants display a normal feeding rate at both one and two weeks of age. (F) Circulating levels of glucose were measured in the hemolymph of *sir2* mutants at one week of age after 24 hours on 8% yeast 15% sugar media, demonstrating hyperglycemia.
(PDF)

S2 Fig Glucose metabolites accumulate in *sir2* mutants. Gas chromatography-mass spectrophotometry analyses was performed on controls (red) and *sir2* mutants (blue) at two weeks of age. The results of three experimental replicates are presented with the exception of dihydroxyacetone phosphate (DHAP), which was undetectable in the third experimental replicate. Sorbitol, a sugar alcohol derived from glucose, is elevated in *sir2* mutants, as is glucose-1-phosphate, an intermediate in glycogen metabolism. Glycolytic intermediates are also elevated, including glucose-6-phosphate, DHAP, phosphoenolpyruvate, and lactate. **p<0.005, ***p<0.0005.
(PDF)

S3 Fig Glucose-stimulated insulin secretion is unaffected in *sir2* mutants that display reduced insulin signaling. (A) RNA was isolated from controls (*cont*) and *sir2* mutants (*sir2 –*) at two weeks of age following a fasting-refeeding paradigm (–/+ glucose) and analyzed by northern blot hybridization. The reduced expression of the Foxo target gene *4EBP* in response to glucose refeeding is blunted in *sir2* mutants, indicative of reduced insulin signaling. (B,C) The non-normalized results of the ELISA assays shown in Fig 2F and 2G are depicted with the mean and ±SEM indicated. Each data point represents a single biological replicate (n = 10 flies/sample, n = 12–30 samples/group). As reported previously, circulating DILP2 levels increase during early adulthood [23]. This can be seen in both controls and *sir2* mutants between one to two weeks of age (fasted to fed). One week controls: 0.016±0.0017 to 0.027±0.0027, two weeks controls: 0.021±0.0043 to 0.042±0.006, one week *sir2* mutants: 0.0068±0.00093 to 0.016±0.0010, two week *sir2* mutants: 0.012±0.0022 to 0.027±0.0043 (two-way ANOVA p<0.0005 between one and two-week-old controls, p<0.0001 one and two-week-old *sir2* mutants). Controls and mutants show similar fold increases in circulating DILP2 in response to feeding, although *sir2* mutants show a slightly enhanced response: one week controls, 1.9-fold, two week controls, 1.7-fold; one week mutants, 2.4-fold, two week mutants, 2.0-fold (two-way ANOVA p<0.05 between fasted and fed controls, p<0.0001 between fasted and fed *sir2* mutants).
(PDF)

S4 Fig *sir2* RNAi results in a strong loss of gene function, and *sir2* rescue restores gene function. (A) *Act-GAL4* was used to drive ubiquitous expression of either *mCherry* (cont) or *sir2 UAS-RNAi* transgenes. Northern blot analysis of RNA isolated from females at two weeks of age following a fasting-refeeding paradigm (–/+ glucose), reveals no detectable *sir2* mRNA upon *sir2* RNAi. (B) GAL4 drivers for the fat body (*r4-GAL4*), muscle (*mef2-GAL4*), or IPCs (*dilp2-GAL4*) were used to express wild-type *UAS-sir2* in an otherwise *sir2* mutant background (black bars), with the GAL4 drivers alone in the mutant background as controls (white bars). Triglycerides were measured in extracts from these animals at two weeks of age and normalized to soluble protein levels (n = 6–15 for each group). Specific expression of wild-type *sir2* in the fat body of *sir2* mutants, but not in the muscle or IPCs, is sufficient to rescue the obese phenotype. (C) Triglycerides are reduced below those of both controls and *sir2* mutants when *UAS-sir2* is expressed in the fat body using the *r4-GAL4* driver (n = 5 for each group).
(PDF)

S5 Fig Sir2 regulates genes involved in metabolism and innate immunity. Gene ontology categories were derived from the *sir2* RNA-seq dataset using the online program DAVID [25]. Categories are divided into “Biological Process” and “Molecular Function”, for both the down-regulated and up-regulated genes. Only the top categories are listed and multiple identical categories are represented by a single entry. The down-regulated genes primarily fall into categories consisting of catabolic enzymes, represented by peptidases, mannosidases, and lipases. Up-regulated genes mainly fall in the innate immune response and stress-response categories.
(PDF)

S6 Fig Sir2 functions are mediated by its protein target dHNF4. (A) A northern blot hybridization was performed on RNA isolated from two independent replicates of control (cont) and *sir2* mutants (*sir2 –*) at 1 or 2 weeks of age, probed to detect *dHNF4*, *sir2*, and *pepck* mRNA. Levels of *dHNF4* and *pepck* mRNA are reduced in *sir2* mutants, although *pepck* is more severely affected. The ratio of *dHNF4* mRNA levels to *rp49* levels in each sample was quantified using data from three independent experimental replicates. The fold change between these ratios in *sir2* mutants and controls is as follows, representing the mean ± SEM: one week 0.7±0.1 (NS), two weeks 0.6±0.09 (p = 0.05). The ratio of *pepck* mRNA levels to *rp49* levels in each sample was quantified using data from three independent experimental replicates. The fold change between these ratios in *sir2* mutants and controls is as follows, representing the mean ± SEM: one week 0.4±0.009 (p = 0.0002), two weeks 0.6±0.09 (p = 0.052). (B) NCBI BLAST alignment of the region in the *Drosophila* (*Dm*) and human (*Hs*) HNF4 sequence shows the conserved lysine residues that are acetylated by p300/CREB in humans (highlighted in yellow). (C,D) Overexpression of *dHNF4* using two copies of the *dHNF4-GFP-FLAG* transgene in an otherwise wild-type animal (control, white bars) or *sir2* mutants (*sir2 –*, black bars) has no effect on the hyperglycemia (C) or high glycogen levels (D) in mutants. Glucose and glycogen were measured at two weeks of age and are normalized to soluble protein levels (n = 6 samples per group). *p<0.05, **p<0.005.
(PDF)

S1 Table List of genes from RNA-seq that display differential abundance between *sir2* mutants and matched controls, meeting a cutoff of a Log2 ratio ± 0.585 (± 1.5 fold) and p-value <0.05. (XLSX)
