# Supplementary figures and images for: Sir2 Acts through Hepatocyte Nuclear Factor 4 to maintain insulin Signaling and Metabolic Homeostasis in Drosophila
Source: PLoS Genet. 2016 Apr 8;12(4):e1005978. doi: 10.1371/journal.pgen.1005978 (PMC4825955; doi:10.1371/journal.pgen.1005978)

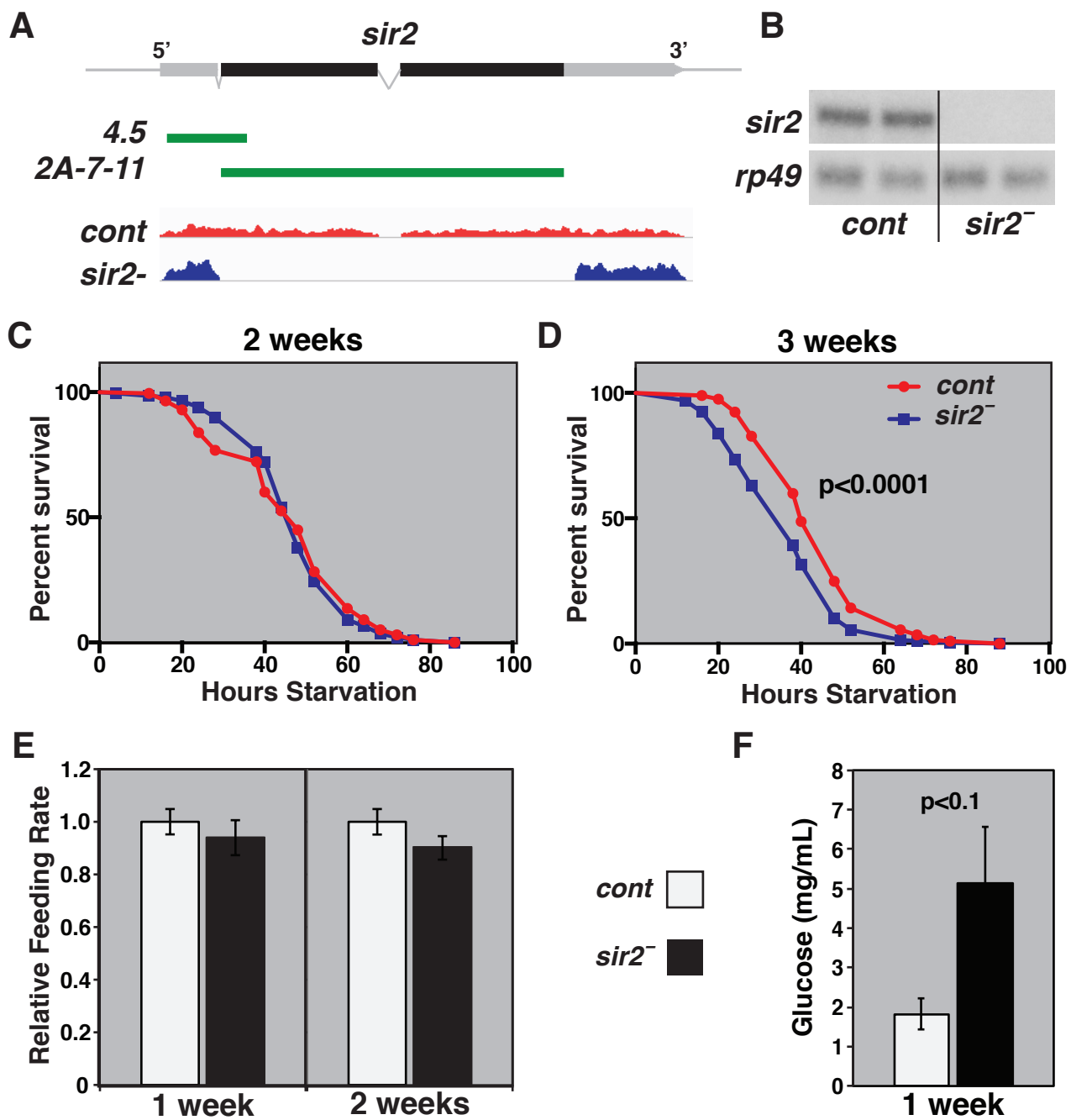

Supplemental Figure 1

Supplement: S1 Fig — (A) A gene model for sir2 is shown, with the coding region in black and non-coding regions in gray. The regions deleted in the sir22A-7-11 and sir24.5 alleles are shown in green. Expression of sir2 from controls (red) and sir2 mutants (blue) was determined by RNA-seq analysis, with the reads assembled using an integrated genomics viewer (IGV). There is no measurable expression of the sir2 coding region in transheterozygous mutants. (B) sir2 transcripts are also not detectable in sir2 mutants by northern blot hybridization, using rp49 mRNA as a loading control. (C,D) The survival of sir2 mutants (sir2 –) on starvation media is similar to that of controls (cont) at two weeks of age (C), but is significantly reduced at three weeks of age (D). (E) sir2 mutants display a normal feeding rate at both one and two weeks of age. (F) Circulating levels of glucose were measured in the hemolymph of sir2 mutants at one week of age after 24 hours on 8% yeast 15% sugar media, demonstrating hyperglycemia. (PDF) [file pgen.1005978.s001.pdf]

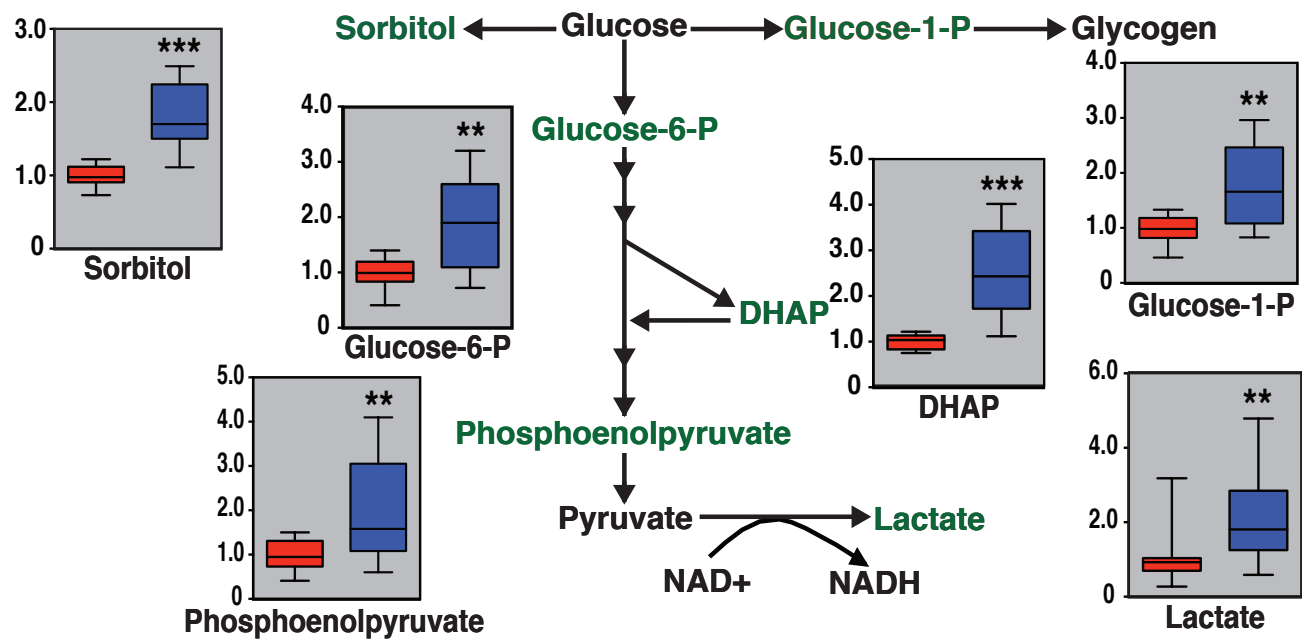

Supplemental Figure 2

Supplement: S2 Fig — Gas chromatography-mass spectrophotometry analyses was performed on controls (red) and sir2 mutants (blue) at two weeks of age. The results of three experimental replicates are presented with the exception of dihydroxyacetone phosphate (DHAP), which was undetectable in the third experimental replicate. Sorbitol, a sugar alcohol derived from glucose, is elevated in sir2 mutants, as is glucose-1-phosphate, an intermediate in glycogen metabolism. Glycolytic intermediates are also elevated, including glucose-6-phosphate, DHAP, phosphoenolpyruvate, and lactate. **p<0.005, ***p<0.0005. (PDF) [file pgen.1005978.s002.pdf]

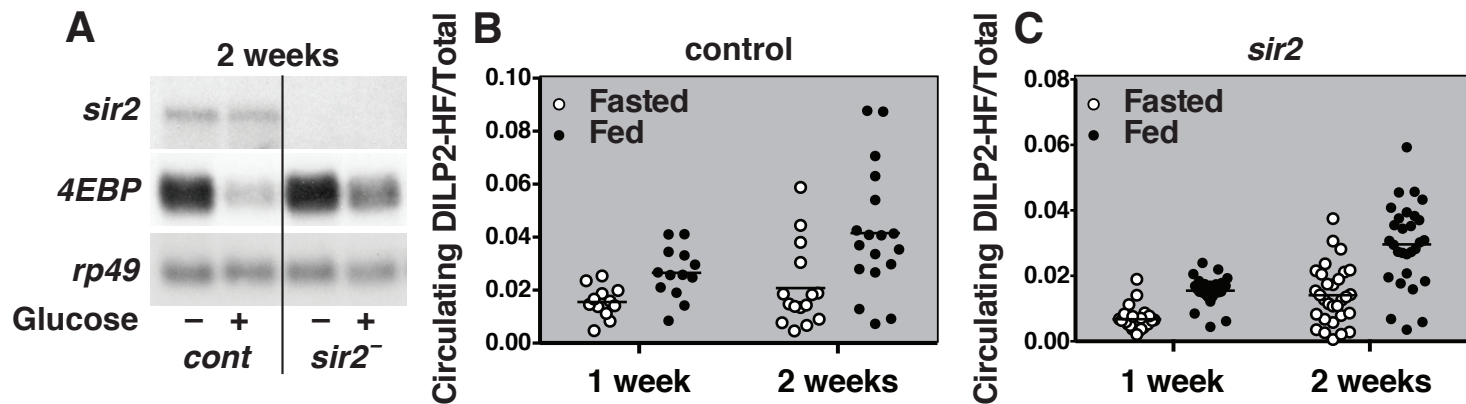

Supplemental Figure 3

Supplement: S3 Fig — (A) RNA was isolated from controls (cont) and sir2 mutants (sir2 –) at two weeks of age following a fasting-refeeding paradigm (–/+ glucose) and analyzed by northern blot hybridization. The reduced expression of the Foxo target gene 4EBP in response to glucose refeeding is blunted in sir2 mutants, indicative of reduced insulin signaling. (B,C) The non-normalized results of the ELISA assays shown in Fig 2F and 2G are depicted with the mean and ±SEM indicated. Each data point represents a single biological replicate (n = 10 flies/sample, n = 12–30 samples/group). As reported previously, circulating DILP2 levels increase during early adulthood [23]. This can be seen in both controls and sir2 mutants between one to two weeks of age (fasted to fed). One week controls: 0.016±0.0017 to 0.027±0.0027, two weeks controls: 0.021±0.0043 to 0.042±0.006, one week sir2 mutants: 0.0068±0.00093 to 0.016±0.0010, two week sir2 mutants: 0.012±0.0022 to 0.027±0.0043 (two-way ANOVA p<0.0005 between one and two-week-old controls, p<0.0001 one and two-week-old sir2 mutants). Controls and mutants show similar fold increases in circulating DILP2 in response to feeding, although sir2 mutants show a slightly enhanced response: one week controls, 1.9-fold, two week controls, 1.7-fold; one week mutants, 2.4-fold, two week mutants, 2.0-fold (two-way ANOVA p<0.05 between fasted and fed controls, p<0.0001 between fasted and fed sir2 mutants). (PDF) [file pgen.1005978.s003.pdf]

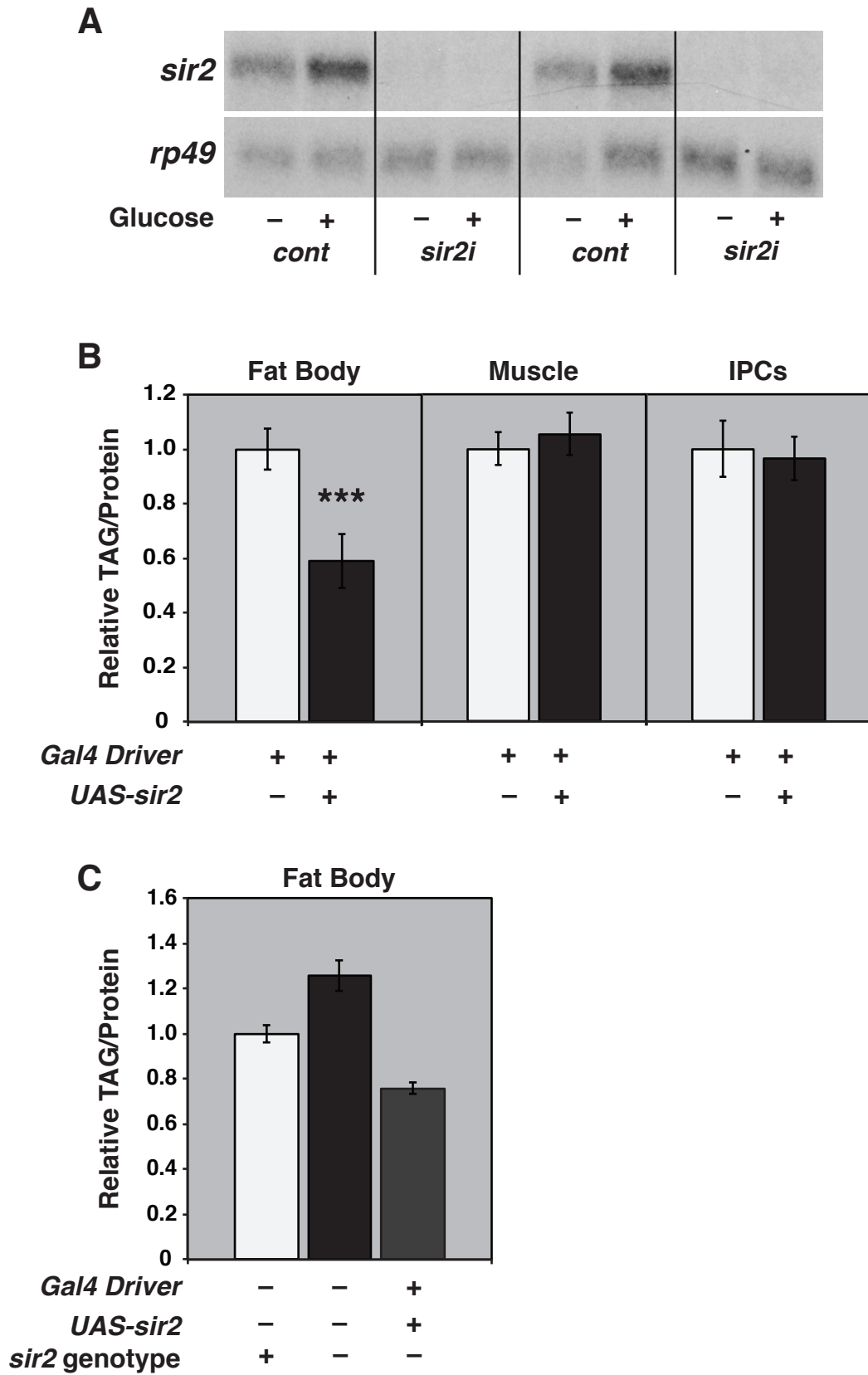

Supplemental Figure 4

Supplement: S4 Fig — (A) Act-GAL4 was used to drive ubiquitous expression of either mCherry (cont) or sir2 UAS-RNAi transgenes. Northern blot analysis of RNA isolated from females at two weeks of age following a fasting-refeeding paradigm (–/+ glucose), reveals no detectable sir2 mRNA upon sir2 RNAi. (B) GAL4 drivers for the fat body (r4-GAL4), muscle (mef2-GAL4), or IPCs (dilp2-GAL4) were used to express wild-type UAS-sir2 in an otherwise sir2 mutant background (black bars), with the GAL4 drivers alone in the mutant background as controls (white bars). Triglycerides were measured in extracts from these animals at two weeks of age and normalized to soluble protein levels (n = 6–15 for each group). Specific expression of wild-type sir2 in the fat body of sir2 mutants, but not in the muscle or IPCs, is sufficient to rescue the obese phenotype. (C) Triglycerides are reduced below those of both controls and sir2 mutants when UAS-sir2 is expressed in the fat body using the r4-GAL4 driver (n = 5 for each group). (PDF) [file pgen.1005978.s004.pdf]

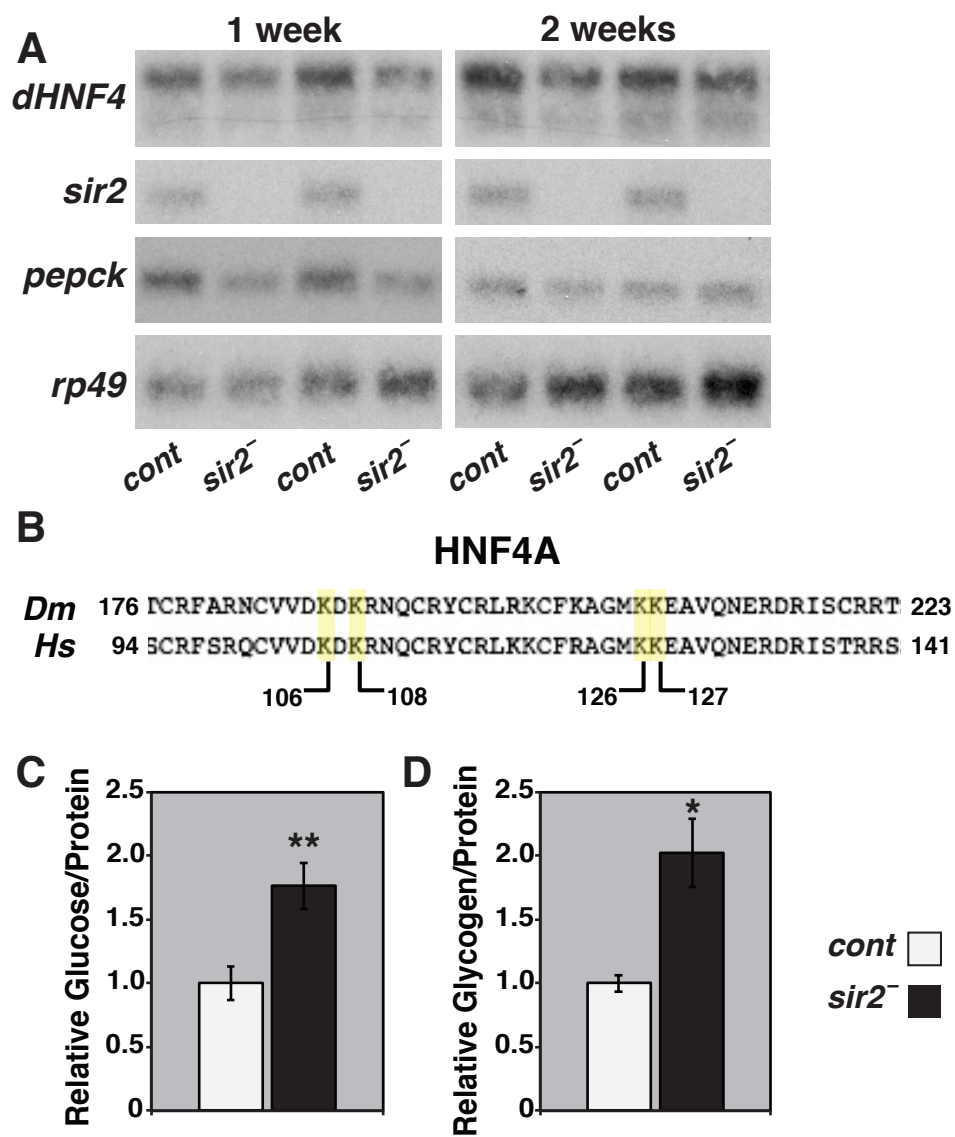

Supplemental Figure 6

Supplement: S6 Fig — (A) A northern blot hybridization was performed on RNA isolated from two independent replicates of control (cont) and sir2 mutants (sir2 –) at 1 or 2 weeks of age, probed to detect dHNF4, sir2, and pepck mRNA. Levels of dHNF4 and pepck mRNA are reduced in sir2 mutants, although pepck is more severely affected. The ratio of dHNF4 mRNA levels to rp49 levels in each sample was quantified using data from three independent experimental replicates. The fold change between these ratios in sir2 mutants and controls is as follows, representing the mean ± SEM: one week 0.7±0.1 (NS), two weeks 0.6±0.09 (p = 0.05). The ratio of pepck mRNA levels to rp49 levels in each sample was quantified using data from three independent experimental replicates. The fold change between these ratios in sir2 mutants and controls is as follows, representing the mean ± SEM: one week 0.4±0.009 (p = 0.0002), two weeks 0.6±0.09 (p = 0.052). (B) NCBI BLAST alignment of the region in the Drosophila (Dm) and human (Hs) HNF4 sequence shows the conserved lysine residues that are acetylated by p300/CREB in humans (highlighted in yellow). (C,D) Overexpression of dHNF4 using two copies of the dHNF4-GFP-FLAG transgene in an otherwise wild-type animal (control, white bars) or sir2 mutants (sir2 –, black bars) has no effect on the hyperglycemia (C) or high glycogen levels (D) in mutants. Glucose and glycogen were measured at two weeks of age and are normalized to soluble protein levels (n = 6 samples per group). *p<0.05, **p<0.005. (PDF) [file pgen.1005978.s006.pdf]
