# Supplementary material for: Sir2 Acts through Hepatocyte Nuclear Factor 4 to maintain insulin Signaling and Metabolic Homeostasis in Drosophila
Source: PLoS Genet. 2016 Apr 8;12(4):e1005978. doi: 10.1371/journal.pgen.1005978 (PMC4825955; doi:10.1371/journal.pgen.1005978)
Supplement: S5 Fig — Gene ontology categories were derived from the sir2 RNA-seq dataset using the online program DAVID [25]. Categories are divided into “Biological Process” and “Molecular Function”, for both the down-regulated and up-regulated genes. Only the top categories are listed and multiple identical categories are represented by a single entry. The down-regulated genes primarily fall into categories consisting of catabolic enzymes, represented by peptidases, mannosidases, and lipases. Up-regulated genes mainly fall in the innate immune response and stress-response categories. (PDF) [file pgen.1005978.s005.pdf]

DOWN	Category	# Genes	p-value	Benjamini
Biological Process	Proteolysis	47	4.80E-13	2.50E-10
	Oxidation reduction	39	1.90E-10	5.10E-08
	Defense response	16	1.60E-06	2.80E-04
	Aminoglycan metabolic process	11	5.10E-04	6.60E-02
	Xenobiotic metabolic process	4	6.00E-04	6.10E-02
	Mannose metabolic process	4	8.40E-04	7.20E-02
	Polysaccharide metabolic process	11	9.20E-04	6.80E-02
Molecular Function	Serine-type peptidase activity	32	2.30E-11	6.40E-09
	Endopeptidase activity	39	3.40E-11	3.10E-09
	Peptidase activity	47	4.90E-11	3.40E-09
	Serine-type endopeptidase activity	30	5.50E-11	3.00E-09
	Peptidase on L-amino acid peptides	45	9.50E-11	4.30E-09
	Carbohydrate binding	20	2.70E-07	1.10E-05
	Lipase activity	11	8.80E-05	3.00E-03
	Heme binding	13	2.00E-04	6.00E-03

UP	Category	# Genes	p-value	Benjamini
BP	Defense response	10	2.20E-07	2.70E-05
MF	Glutathione transferase activity	4	6.50E-04	5.20E-02

Supplemental Figure 5
